# The Prevalence and Contributing Factors of Burnout Among Anesthesiologists and Intensive Care Unit Staff in Morocco: A Cross-Sectional Analysis

**DOI:** 10.7759/cureus.44956

**Published:** 2023-09-09

**Authors:** Sabah Benhamza, Mohammad Khalayla, Laila Lahlou, Zineb Amine, Mohamed Lazraq, Youssef Miloudi, Abdelhak Bensaid, Najib El Harrar

**Affiliations:** 1 Anesthesia and Critical Care, Ibn Rochd University Hospital Center, Casablanca, MAR; 2 Laboratory of Health Sciences, Faculty of Medicine and Pharmacy, Ibn Zohr University, Agadir, MAR; 3 Anesthesia and Critical Care, Ibn Rochd University Hospital Center, Hassan II University of Casablanca, Casablanca, MAR

**Keywords:** morocco, prevalence, risk factors, anaesthesia and intensive care, burnout

## Abstract

Introduction

Burnout is a common issue in the medical field, particularly in specialties like anaesthesiology and intensive care. It carries significant personal and professional consequences for healthcare providers and can impact the relationship between caregivers and patients. Despite its seriousness, there's been limited research on its causes in North Africa. In this study, our aim was straightforward: we wanted to find out how prevalent burnout is among Moroccan healthcare workers in anaesthesiology and intensive care and identify the main factors contributing to it.

Methods

To achieve this, we conducted a comprehensive multicenter cross-sectional study that included hospitals from different regions of Morocco. We focused on anesthesiologists and nurse anesthetists currently practicing in these settings. We measured burnout using the French version of the Maslach Burnout Inventory.

Results

We distributed 500 questionnaires and received and analyzed 396 of them, accounting for an 84% response rate. The results were striking: 48% of participants experienced high emotional exhaustion, 43.2% had a significant level of depersonalization, and 21% exhibited a low sense of personal accomplishment. When we looked at various factors, such as age, income, on-call duties, years of experience, and work location, our analysis showed statistically significant differences in all three dimensions of burnout. In our more complex multivariate analysis, we found that the risk factors for all three dimensions of burnout were practicing for 5 to 15 years and participating in on-call rotations. Surprisingly, practicing for over 25 years seemed to be a protective factor against all dimensions of burnout.

Conclusion

Our study clearly indicates that burnout is a shared issue among healthcare professionals in anaesthesiology and intensive care units in Morocco. Importantly, we've pinpointed specific risk factors that should be the foundation for a national strategy to prevent burnout in these critical healthcare sectors.

## Introduction

Burnout (BO), also known as professional exhaustion syndrome, is an individual response to chronic work-related stress that develops progressively and can eventually become chronic, leading to adverse health effects. It results in a decline in the quality of life for caregivers and can strain the doctor-patient relationship. Burnout is characterized by three dimensions: depersonalization, burnout, and personal accomplishment. Healthcare professionals, including doctors and nurses, often grapple with pain, death, stress, and medical-legal issues, which generate intense emotions and chronic stress, contributing to the widespread occurrence of burnout in medical settings [1]. This issue is even more pronounced in the fields of anaesthesiology and intensive care units, where professionals routinely encounter complex cases with limited prognostic outcomes. Despite the profound consequences it entails, burnout remains an underexplored condition in the Maghreb countries, particularly in Morocco. The primary goal of our study is to ascertain the prevalence of burnout and its associated risk factors among nurse anaesthetists and anesthesiologists, with the aim of proposing preventive measures.

A recent research has reported wide-ranging prevalence estimates for burnout, ranging from 6% to 33%, across various dimensions of burnout in a study encompassing 22,177 general practitioners from 29 different countries, as detailed in a recently published review [2].

The main objective of our study is to determine the prevalence of burnout syndrome among Moroccan doctors and nurses working in anaesthesia and intensive care units and to identify potential determinants of burnout.

## Materials and methods

Study design and framework

This study employed a multicenter cross-sectional survey conducted through questionnaires over a period of nine months, spanning from April to December 2019. The survey encompassed all hospitals in Morocco and specifically targeted two distinct groups of healthcare professionals within the fields of anaesthesiology and critical care. The first group comprised doctors, including professors from various university hospital centers (UHCs), residents, and physicians stationed at provincial (PHC) or regional hospital centers (RHC). The second group consisted of nurse anaesthetists working in either the operating room or the intensive care unit.

Study population and sampling

All active doctors and nurses assigned to intensive care units or working in operating rooms at the time of the study were included. Exclusions were made for trainees, anesthesiologists, and/or nurse anaesthetists not working in these specific units, individuals on leave during the study period, doctors temporarily assigned to the intensive care unit, participants who declined to partake in the study, and incomplete questionnaires. Sampling was accomplished using a probability proportional to staff size approach within each region. For a statistical power of 95%, our sample size was determined to be 160 doctors and 66 nurses.

Data collection and variables studied

Our survey utilized a questionnaire divided into five sections. The first section collected socio-demographic information, while the second focused on professional data, including job roles, practice locations, and interactions with hospital administration. The third section gathered personal data. The fourth section assessed knowledge about burnout (BO), and the fifth section administered the French version of the Maslach Burnout Inventory (MBI) scale. The MBI was chosen for its wide use in measuring burnout, and because our study population was proficient in both French and Arabic, there was no need for translation. The MBI evaluates burnout across three dimensions: emotional exhaustion (EE, 9 items), depersonalization (DP, 5 items), and personal accomplishment at work (PA, 8 items). Each item is rated on a six-point scale from 0 ("never") to 6 ("every day"). The score for each dimension is the sum of the ratings for its respective items, allowing classification into three severity levels (low, moderate, or high). Low EE scores were ≤16, moderate EE scores were 17-26, and high EE scores were >27. Low DP scores were ≤6, moderate DP scores were 7-12, and high DP scores were >13. Low PA scores were ≥37, moderate PA scores were 31-36, and high PA scores were ≤30. A high score in EE or DP or a low score in PA indicated a state of burnout. Based on the number of affected dimensions, different severity stages were identified: low (one dimension affected), moderate (two dimensions affected), and severe (all three dimensions affected). The questionnaire underwent a preliminary test by an intern assigned to an intensive care unit at the UHC of Ibn Rochd in Casablanca before distribution. The questionnaires were provided to the head nurses of various intensive care units or operating centers following approval from department heads. After approximately 15 days, the questionnaires were collected. While paper surveys were employed in five of the 12 Moroccan regions (Casablanca-Settat, Drâa Tafilalet, Eastern, Tangier-Tetouan-Al Houceima, and Béni Mellal-Khénifra), the rest of the regions utilized electronic submissions.

Data analysis and statistical methods

The data collected underwent analysis using SPSS v.13.0 (SPSS Inc., Chicago, IL, USA). Initially, qualitative variables were converted into quantitative ones with intervals to facilitate analysis. A quantitative assessment followed, presenting variables in numbers and percentages. Subsequently, a bivariate analysis was conducted to explore the relationship between various parameters and each dimension of burnout (low PA, high EE, or high DP). The Chi-square test was used when counts were equal to or exceeded 5; otherwise, Fisher's exact test was applied. In both cases, significance was set at p<0.05. Finally, a multivariate analysis was performed using logistic regression for each of the three dimensions of burnout, adjusting for EE, DP (high), or PA (low).

Ethical considerations

A standardized questionnaire was administered to all participants, who had the option to complete the strictly anonymous questionnaires at their discretion.

## Results

Participants

We distributed 500 questionnaires, and 423 were successfully collected, resulting in an 84.6% response rate. Out of these, 396 questionnaires (93.6%) met our inclusion criteria, while 27 were excluded (6.4%). Of the participants, 170 (42.9%) were doctors, and 226 (57.1%) were nurses.

Socio-demographic characteristics of the participants

Among the 170 physicians, 59% were specialists practicing in PHC, RHC, or the private sector, 31% were residents in training, and 10% held the position of a professor. Within the group of 226 nurses, 48% were nurse anaesthetists working in the operating room, while the remaining nurses worked in the intensive care unit. Geographically, the majority of responses came from the Casablanca-Settat region (25%), followed by 14.9% from Rabat-Salé-Kenitra, 13.9% from Marrakech-Safi, 12.1% from the Fez-Meknes region, 10.1% from the Oriental region, 7.1% from the Souss-Massa region, 5.1% from Tangier-Tetouan-Al Hoceima, 3% from the Béni Mellal-Khénifra region, 3% from Draa-Tafilalt, 2% from the Laâyoune-Saguia al Hamra region, 2% from Ed Dakhla-Oued ed Dahab, and 1.8% from the Guelmim-oued Noun region. Notably, 83% of both doctors and nurses indicated familiarity with the concept of burnout (BO). Further details regarding participants' characteristics based on the first three sections of our questionnaire can be found in Table 1.

**Table 1:** Socio-demographic, personal and professional characteristics of the participants

Characteristics	Participants, N (%)		
	Doctors (n = 170)	Nurses (n = 226)	Total caregivers (n = 396)
Age (years)			
≤30	106 (62.35)	135 (59.73)	241 (60.90)
>30 - ≤40	34 (20.00)	67 (29.65)	101 (25.50)
>40 - ≤55	13 (7.65)	9 (3.98)	22 (5.60)
>55	17 (10.00)	15 (6.64)	32 (8.10)
Female sex	56 (32.94)	155 (68.58)	211 (53.30)
Family situation			
Singles	94 (55.29)	127 (56.19)	221 (55.80)
Married	67 (39.41)	91 (40.27)	158 (39.90)
Divorced	9 (5.29)	8 (3.54)	17 (4.30)
No dependent children	134 (78.82)	186 (82,30)	320 (80,80)
Number of years of practice			
Less than 5 years	85 (50.00)	88 (38.90)	173 (43.70)
Between 5 and 15 years	50 (29.40)	101 (44.70)	151 (38.10)
Between 16 and 25 years	9 (5.30)	15 (6.60)	24 (6.10)
More than 25 years	26 (15.30)	22 (9,70)	48 (12,10)
Career choice			
Deliberate	123 (72.35)	202 (89,38)	325 (82,07)
Imposed	47 (27.65)	24 (10.62)	71 (17.93)
Place of practice choice			
Deliberate	164 (96.47)	139 (61.50)	303 (76.52)
Imposed	6 (3.53)	87 (38.50)	93 (23.48)
Structure of practice			
Peripheral hospital	79 (46.47)	101 (44.69)	180 (45.50)
Regional hospital	18 (10.59)	24 (10.62)	42 (10.60)
University hospital	45 (26.47)	64 (28.32)	109 (27.50)
Liberal sector	24 (14.12)	36 (15.93)	60 (15.20)
Non-profit organization	4 (2.35)	1 (0.44)	5 (1.30)
Number of working hours per week (including on-call duty)			
Less than 15	8 (4.71)	32 (14.16)	40 (10.10)
Between 15 and 35	3 (1.76)	48 (21.24)	51 (12.88)
Between 35 and 50	44 (25.88)	97 (42.92)	141 (35.61)
More than 50	115 (67.56)	49 (21.68)	164 (41.41)
Number of free weekends per month			
None	0 (0.00)	53 (23.45)	53 (13.38)
1 only	0 (0.00)	18 (7.96)	18 (4.55)
2	110 (64.71)	53 (23.45)	163 (41.16)
3	58 (34.12)	41 (18.14)	99 (25.00)
All	2 (1.18)	61 (26.99)	63 (15.91)
Shift system			
On call	128 (75.30)	67 (29.60)	195 (49.20)
Shift in Hospital	42 (24.70)	159 (70.40)	201 (50.80)
Number of shifts per month			
None	0 (0.00)	29 (12.83)	29 (7.32)
Less than 5 shifts	27 (15.88)	42 (18.58)	69 (17.42)
From 5 to 10 shifts	109 (64.12)	67 (29.65)	176 (44.44)
More than 10 shifts	34 (20.00)	88 (38.94)	122 (30.81)
Lack of rest after shift	155 (91.18)	116 (51.33)	125 (31.60)
Get the vacation			
Easy	56 (32.90)	73 (32.30)	129 (32.6)
Difficult	114 (67.10)	153 (67.70)	267 (67.40)
Workload estimate			
Important	147 (86.47)	128 (56.64)	275 (69.40)
Middle	23 (13.53)	91 (40.27)	114 (28.80)
Minimal	0 (0.00)	7 (3.10	7 (1.80)
Estimation of salary			
Adapted	9 (5.29)	32 (14.16)	41 (10.35)
Non-adapted	161 (94.71)	194 (85.84)	355 (89.39)
Estimation of time spent with family			
Sufficient	38 (22.35)	58 (25.66)	96 (24.24)
Insufficient	132 (77.65)	168 (74.34)	300 (75.76)
The presence of entertainment (Yes)	128 (75.29)	154 (68.14)	282 (71.20)
Continuing education (Yes)	53 (31.18)	60 (26.55)	113 (29.50)
Presence of toxic habits	63 (37.06)	26 (11.50)	89 (22.50)
Nature of toxic habits			
Tobacco	55 (32.43)	122 (54.00)	177 (44.70)
Alcohol	51 (29.73)	77 (34.00)	128 (32.32)
Cannabis	32 (18.92)	22 (10.00)	54 (13.63)
Anesthetic products	14 (8.11)	5 (2.00)	19 (4.80)
Psychotropics	18 (10.81)	0 (0.00)	18 (4.55)
Presence of sleep disturbances	66 (38.82)	103 (45.58)	169 (42.70)
Presence of suicidal ideation	0 (0.00)	1 (0.44)	1 (0.25)

Prevalence of the three dimensions of burnout (BO)

The final section of our questionnaire focused on the Maslach Burnout Inventory (MBI). In our study, we found that 21% of participants reported low Personal Accomplishment (PA), 48% experienced high Emotional Exhaustion (EE), and 43.2% exhibited high Depersonalization (DP), as shown in Table 2.

**Table 2:** Distribution of caregivers according to the three dimensions of burnout (BO)

Dimensions of the BO	Participants, N (%)		
	Doctors (n = 170)	Nurses (n = 226)	Total caregivers (n = 396)
Emotional exhaustion			
Down	47 (27.60)	58 (25.70)	105 (26.50)
Moderate	39 (22.90)	62 (27.40)	101 (25.50)
High	84 (49.40)	106 (46.90)	190 (48.00)
Depersonalization			
Down	53 (31.20)	41 (18.10)	94 (23.70)
Moderate	50 (29.40)	81 (35.80)	131 (33.10)
High	67 (39.40)	104 (46.00)	171 (43.20)
Personal accomplishment			
Down	36 (21.20)	47 (20.80)	83 (21.00)
Moderate	38 (22.40)	35 (15.50)	73 (18.40)
High	96 (56.50)	144 (63.70)	240 (60.60)

Factors associated with dimensions of burnout (BO)

Our bivariate analysis unveiled statistically significant differences between the three dimensions of burnout and various factors including age groups, salary, the on-call system, years of practice, and practice location. For a detailed breakdown, see Table 3.

**Table 3:** Univariate analysis of the different characteristics according to high levels of EE and DP and a low level of PA in caregivers.

	Emotional exhaustion (EE)	Depersonalization (DP)	Personal Accomplishment (PA)
Factors studied	p	p	p
Age groups	<0.0001	<0.0001	<0.0001
Gender	0.263	0.807	0.883
Marital status	0.655	0.64	0.666
Have children	0.093	0.277	0.57
Weakly working hours	0.088	0.193	0.013
Number of shifts	0.055	0.296	0.995
Lack of rest after shift	0.661	0.044	0.012
Free weekends per month	0.031	0.33	0.764
Difficulty in getting vacation	0.03	0.358	0.08
Hobbies	0.756	0.941	0.496
The formation continues	0.198	0.383	0.056
Workload	0.182	0.415	0.357
Toxic habits	0.747	0.517	0.23
Sleep disorders	0.215	0.21	0.99
Need for psychiatric follow-up	0.726	0.549	0.484
Regret of career choice	0.438	0.762	0.139
Insufficient salary	<0.0001	0.0001	0.0001
Deliberate choice of speciality	0.546	0.541	0.426
Place of practice	0.011	0.043	0.023
Shift system	<0.0001	<0.0001	<0.0001
Number of years of practice	0.005	0.0001	0.0001

Factors associated with burnout (BO)

In our multivariate analysis, several factors were found to be associated with the different dimensions of burnout (EE, DP, and PA).

For the Emotional Exhaustion (EE) dimension, risk factors included individuals between 30 and 40 years old, having less than 25 years of practice, working in a primary healthcare setting (PHC), being part of an on-call system, regretting their career choice, and facing difficulty in taking vacations. Conversely, protective factors encompassed professionals over 40 years old working in a university hospital center (UHC) or regional hospital center (RHC), as detailed in Table 4.

**Table 4:** Multivariate analysis of the different characteristics according to high levels of EE and DP and a low level of PA in caregivers PHC: Primary healthcare center; UHC: University hospital center; RHC: Regional hospital center.

Characteristics	Emotional Exhaustion (EE)		Depersonalization (DP)		Personal accomplishment (PA)	
	OR	IC 95% Lower - upper	OR	IC 95% Lower - upper	OR	IC 95% Lower - upper
Age (years)						
≤30	0.746	0.473-1.177	2.81	1.826-4.32	3.189	2.09-4.87
>30 - ≤40	2.214	1.462-3.352	1.716	1.182-2.50	0.673	0.426-1.06
>40 - ≤55	0.212	0.071-0.634	7.98	1.897-33.58	0.395	0.167-0.94
>55	0.396	0.178-0.878	0.668	0.313-1.43	8.308	3.269-21.11
Have children	0.571	0.344-0.948	1.082	0.979-1.20	1.314	0.793-2.18
Number of years of practice						
Less than 5 years	2.139	1.42-3.23	0.023	0.011-0.50	0.9	0.6-1.35
Between 5 and 15 years	2.405	1.27-4.54	60.729	31.435-117.32	2.15	1.394-3.32
Between 16 and 25 years	1.638	1.09-2.47	0.395	0.348-0.45	0.581	0.533-0.63
More than 25 years	0.681	0.54-0.86	0.509	0.459-0.57	0.1	0.045-0.22
Practice structure						
PHC	2.158	1.442-3.228	1.054	0.707-1.57	2.061	1.274-3.33
RHC	0.409	0.226-0.739	34.046	8.095-143.18	0.559	0.51-0.61
UHC	0.206	0.124-0.343	0.469	0.293-0.75	0.955	0.431-1.64
Liberal sector	0.167	0.108-0.261	0.347	0.184-0.65	1.18	0.6-2.3
Number of working hours per week (including on-call duty)						
Less than 15	0.928	0.834-1.03	0.97	0.871-1.08	1.103	0.766-1.59
Between 15 and 35	1.386	0.863-2.22	0.874	0.543-1.41	0.88	0.547-1.42
Between 35 and 50	1.286	0.891-1.86	1.109	0.768-1.60	1.136	0.706-1.83
More than 50	0.722	0.45-1.16	0.666	0.403-1.10	1.103	0.766-1.59
Number of free weekends per month						
none	1.147	0.643-2.05	0.924	0.514-1.66	0.989	0.547-1.79
1 only	1.375	0.531-3.56	1.055	0.407-2.73	1.316	0.483-3.59
2	0.912	0.611-1.36	1.071	0.715-1.60	0.848	0.563-1.28
3	1.584	1.002-2.51	1.194	0.756-1.89	1.257	0.784-2.02
all	0.485	0.275-0.85	0.719	0.412-1.25	0.911	0.527-1.58
On-call duty	-	3.439-5.2	5.6	0.61-8.69	2.382	1.575-3.60
Number of shifts per month						
None	0.75	0.348-1.61	1.248	0.586-1.23	1.005	0.95-1.06
Less than 5 shifts	0.823	0.548-1.35	0.953	0.741-1.23	0.894	0.59-1.36
From 5 to 10 shifts	0.856	0.694-1.06	1.03	0.881-1.21	1.069	0.491-2.33
More than 10 shifts	1.359	0.886-2.08	1.015	0.66-1.56	1.043	0.892-1.22
Lack of rest after on-call duty	1.099	0.719-1.68	0.787	0.511-1.21	1.766	1.125-2.77
Difficulty in getting vacation	1.595	1.045-2.435	1.113	0.729-1.70	1.543	0.993-2.40
Workload estimate						
Important	1.473	0.956-2.27	0.875	0.569-1.35	1.302	0.843-2.01
Moderate	0.717	0.462-1.11	1.149	0.742-1.78	0.811	0.521-1.26
Minimal	0.428	0.082-2.23	0.987	0.218-4.47	0.481	0.106-2.18
Time spent on continuing education	0.815	0.596-1.11	0.815	0.504-1.32	0.585	0.376-0.91
Regret of career choice	2.074	1.19-3.61	0.895	0.635-1.63	0.792	0.456-1.37
Insufficient salary	1.163	0.773-1.75	2.139	1.416-3.23	1.178	0.445-3.12

In the Depersonalization (DP) dimension, risk factors encompassed individuals under 55 years old, working in an RHC, having a total practice duration between 5 and 25 years, receiving an insufficient salary, and being part of an on-call system. Protective factors for this dimension included having less than 5 years or more than 16 years of practice, also outlined in Table 4.

Lastly, concerning the Personal Accomplishment (PA) dimension, risk factors involved individuals under 30 or over 55 years old, lacking adequate rest after on-call duties, practicing in a PHC, being part of an on-call duty system, having a practice duration between 5 and 15 years, and receiving an insufficient salary. Protective factors consisted of professionals aged between 40 and 55 years, working in an RHC or a UHC, or having continuous training exceeding 16 years, as shown in Table 4.

## Discussion

Burnout (BO) is a serious condition with far-reaching consequences for individuals and institutions. At the individual level, BO can lead to detrimental behaviors, including addiction and, in severe cases, even suicide. This stems from the individual's struggle to cope with errors, which are a natural part of human cognitive functioning [3]. Normally, errors are manageable, but when an individual experiences BO, problem-solving becomes significantly more challenging, leading to a decline in professional performance [3-5]. On an institutional level, BO can result in job changes or even abandonment of careers. This issue is particularly acute in the medical field, where there is a shortage of professionals, especially in specialties like anaesthesia and intensive care units.

Our study covered various regions in Morocco, achieving an 84.6% response rate, which is representative of our target population. However, one significant challenge we encountered during the study was defining BO, as its definition varies among studies. Some authors describe it based on a single pathological score within one of the three dimensions [6], while others classify it based solely on high scores in Emotional Exhaustion (EE) or Depersonalization (DP) [6-8].

BO prevalence can vary across studies, depending on whether all dimensions are assessed separately. In our study, we observed concerning rates: 21% reported low Personal Accomplishment (PA), 48% experienced high Emotional Exhaustion (EE), and 43.2% exhibited high Depersonalization (DP). These rates are notably higher than those reported in European countries like Portugal [8]. Another study by Morais et al. found that 50-60% of anesthesiologists suffered from BO [9]. A Tunisian study reported rates of 26.68% for PA, 31.44% for EE, and 31.44% for DP, which are similar to our findings. Additionally, our study calculated a BO rate of 83% for physicians and 95% for nurse anaesthetists [8].

We also noted that professionals aged between 30 and 40 were more susceptible to EE and DP dimensions, while those over 40 were less affected. This could be attributed to the fact that a majority of respondents (45%) worked in peripheral hospitals, which pose their own risks. These hospitals often treat younger patients and lack the same resources as larger, urban hospitals. Older professionals tend to work in university hospital centers (UHC), regional hospital centers (RHC), or in private practice, which may explain their lower vulnerability to BO. This difference might be due to age itself or the experience they have gained in managing stress and conflicts, making them more resilient to BO. To mitigate BO, it's essential to provide support and training for young doctors and nurses and reduce their working hours in favor of support and training activities [9,10].

In Morocco, the on-call system compensates for the shortage of anesthesiologists but emerges as a risk factor for all three dimensions of BO. The on-call system mandates that a doctor be available for 16 hours per day on working days and 24 hours on holidays and weekends when the number of anesthesiologists in a hospital is insufficient. Article 3 of the Moroccan law governing the on-call system requires the presence of the carer within the territory of the prefecture and/or the province to which the care unit he is assigned belongs, as well as the obligation to join the unit immediately after being notified [9]. This system is incompatible with a specialty like anaesthesia-resuscitation, where urgent interventions are often required. It's likely responsible for weekly working hours exceeding 50. We recommend transitioning to a residential on-call duty system with compensatory rest and increasing the recruitment of healthcare professionals, especially in peripheral hospitals. These changes would contribute to reducing BO among healthcare providers.

The lack of compensatory rest after shifts was a risk factor only in the PA dimension, while difficulty obtaining leave was correlated with EE. Nurses can benefit from better distribution of human resources to allow for rotation among various services, while doctors can benefit from more effective resource management at regional and national levels. A public-private partnership could also help address the shortage of critical care specialists in both large and small cities. Furthermore, healthcare workers' salaries should be reviewed to account for their excessive workload.

While our study did not establish a direct correlation between BO and toxic habits, suicide attempts, or sleep disorders, it did reveal concerning findings. About 37% of doctors and 11% of nurses reported toxic habits, including the use of anesthetic products. Additionally, 0.44% of nurses reported having suicidal thoughts, and nearly half of the participants experienced sleep disorders. These results show distinctive evidence to what is described in several studies [11-13]; they are still alarming. These issues are likely underestimated because they are considered taboo subjects in Muslim society, especially within the professional environment. Therefore, it's essential to establish psychiatric care in each region and raise awareness about BO to prevent suicidal acts, especially among nurses who may not be familiar with the concept (only 74% of nurses in our study were aware of BO, compared to 96% of doctors).

Two major limitations of our study should be addressed in future research. Firstly, the study encompassed all healthcare workers and did not specifically focus on doctors or nurses individually. Secondly, there was a disparity in participant numbers, with a higher percentage of nurses than doctors.

## Conclusions

Burnout is a growing concern that affects individuals, organizations, and society as a whole. Individuals experiencing burnout often suffer from a persistent sense of exhaustion that goes beyond their official working hours. Even the mere thought of work before starting the day can leave them feeling drained and depleted of energy. It's essential to understand that burnout is not an inevitable condition; it can be prevented before it develops and effectively addressed during its progression. However, interventions often focus on individual factors rather than addressing the root organizational causes, such as excessive workloads or role ambiguity. Healthcare institutions should proactively assess the well-being of their staff on a regular basis, using both quantitative and qualitative methods, and consider it a crucial performance indicator.

This study underscores the pressing need for specific national actions and preventative strategies aimed at reducing burnout (BO) within the anaesthesia-resuscitation field. These measures are vital to protect the well-being of our healthcare professionals and to prevent potentially severe consequences on both individual and institutional levels.

## References

[1] Letonturier P (2008). Psychiatrie - Le Syndrome D’épuisement Professionnel du Soignant, Une Nouvelle Maladie Professionnelle. https://www.em-consulte.com/article/102212/psychiatrie-le-syndrome-d-epuisement-professionnel.

[2] Shen X, Xu H, Feng J, Ye J, Lu Z, Gan Y (2022). The global prevalence of burnout among general practitioners: a systematic review and meta-analysis. Fam Pract.

[3] Gambarana C, Masi F, Tagliamonte A, Scheggi S, Ghiglieri O, De Montis MG (1999). A chronic stress that impairs reactivity in rats also decreases dopaminergic transmission in the nucleus accumbens: a microdialysis study. J Neurochem.

[4] Basner RC (2005). Shift-work sleep disorder--the glass is more than half empty. N Engl J Med.

[5] Larouche LM (1985). Manifestations cliniques du “burn out” chez les médecins. Santé mentale au Québec.

[6] Shanafelt TD, Bradley KA, Wipf JE, Back AL (2002). Burnout and self-reported patient care in an internal medicine residency program. Ann Intern Med.

[7] Schaufeli WB, Van Dierendonck D (1995). A cautionary note about the cross-national and clinical validity of cut-off points for the Maslach Burnout Inventory. Psychol Rep.

[8] Mhamdi S, Nakhli MS, Kahloul M (2018). Burnout prevalence in Tunisian anesthesia and intensive care units [Article in French]. Pan Afr Med J.

[9] Morais A, Maia P, Azevedo A, Amaral C, Tavares J (2006). Stress and burnout among Portuguese anaesthesiologists. Eur J Anaesthesiol.

[10] Ministry of Economy, Finance and Administration Reform, Kingdom of Morocco (2007). Décret n°2-06-623 du 24 rebia I 1428 ( 13 1vril 2007) relative à indemnité de gardiennage et de service obligatoire réalisé par certains fonctionnaires du ministère de la santé et les agents des centres hospitaliers. https://www.em-consulte.com/article/102212/psychiatrie-le-syndrome-d-epuisement-professionnel.

[11] Soler JK, Yaman H, Esteva M (2008). Burnout in European family doctors: the EGPRN study. Fam Pract.

[12] Chen KY, Yang CM, Lien CH, Chiou HY, Lin MR, Chang HR, Chiu WT (2013). Burnout, job satisfaction, and medical malpractice among physicians. Int J Med Sci.

[13] Cathébras P, Begon A, Laporte S, Bois C, Truchot D (2004). Burn out among French general practitioners [Article in French]. Presse Med.

